# The marketing plan and outcome indicators for recruiting and retaining parents in the HomeStyles randomized controlled trial

**DOI:** 10.1186/s13063-017-2262-3

**Published:** 2017-11-15

**Authors:** Carol Byrd-Bredbenner, Colleen Delaney, Jennifer Martin-Biggers, Mallory Koenings, Virginia Quick

**Affiliations:** Rutgers, The State University of New Jersey, 26 Nichol Avenue, New Brunswick, NJ 08901 USA

**Keywords:** Recruitment, Retention, Parents, Preschool, Children, Marketing

## Abstract

**Background:**

Despite the critical importance of successful recruitment and retention to study integrity, reporting of recruitment and retention strategies along with factors associated with successful recruitment and retention of participants in health-related interventions remain rare, especially for health and obesity prevention programs. Thus, the purpose of this article is to retrospectively examine the recruitment and retention marketing plan used in the online HomeStyles randomized controlled trial (RCT) and discuss outcomes associated with completion of the intervention.

**Methods:**

The HomeStyles RCT is an online intervention developed to motivate parents of young children to gain the skills and self-confidence needed to shape home environments and lifestyles to be protective against childhood obesity. Using the seven Ps of services marketing (i.e., people, place, product, physical evidence, price, promotion, and process), a comprehensive and systematic plan for recruitment and retention was implemented and outcomes assessed.

**Results:**

A total of 489 parents with a young child aged 2 to < 6 years were eligible to participate, a final capture rate of 33%. Only 23% of Hispanic participants chose to use the Spanish-language version of HomeStyles intervention materials, below the demand anticipated. However, Hispanic enrollment overall was substantially higher than the U.S. population proportion (i.e., 17%). The number of participants prematurely leaving the study was similar in both treatment groups, indicating attrition was not differential. Completers reported high satisfaction of HomeStyles, using a 1–5 scale (strongly disagree to strongly agree) on guide attractiveness, interestingness, and usefulness. Despite all the retention efforts, the average monthly recruitment accrual rate of ~ 33 eligible enrolled participants at baseline (i.e., 489 participants/15-month recruitment period), declined to ~ 18, 11, 9, and 8 remaining recruited participants/month at midpoint, post, follow-up, and long-term follow-up surveys, respectively. In general, survey completers were significantly more likely to be female and perceived their child’s health status to be better, and they were significantly less likely to be restrictive of their child’s food intake.

**Conclusions:**

The findings of the present study highlight the need for far-reaching, concentrated, and varied recruitment strategies; sufficient time in the research plan for recruitment and retention activities; and creative, tireless, flexible, persistent project staff for health-related interventions.

## Background

According to the Institute of Medicine, “[F]amilies play a central role in childhood obesity prevention.… Innovative approaches are needed to provide families with relevant obesity prevention information, particularly information that is practical, that is easily implemented, and that does not judge or lecture parents” [1]^, p. 343^. To address this need, the HomeStyles program was developed to help parents shape home environments and lifestyle practices to be more supportive of optimal child health and weights [2–5]. A randomized controlled trial (RCT) of this online, theory-driven, evidence-based intervention was conducted between 2014 and 2016 [6].

The home environment plays a central role in establishing children’s weight-related patterns (e.g., eating and physical activity behaviors) [7–9]. Thus, parents were the target audience of this intervention because they create the home lifestyle environment (e.g., set expectations and routines, such as participation in family mealtimes), are the household gatekeepers (e.g., decide which foods are available in the home), establish family policies (e.g., hours children are permitted to watch television), and serve as children’s role models (e.g., demonstrate weight-related behaviors, such as eating and physical activity) [10–21]. Parents also must have more opportunities to develop the knowledge, cognitions, skills, and behaviors needed to prevent childhood obesity and the motivation to incorporate them in their hectic lifestyles [1].

Attracting individuals to participate in health-related intervention studies and retaining them for the duration of the study is critical to establishing the usefulness and acceptability of interventions, maintaining internal and external validity, and preserving statistical power [22–24]. Factors that predict the success of enrolling and retaining study participants remain understudied for all population groups, including parents and families with young children. Additionally, knowledge of factors affecting recruitment and retention by program type, such as health prevention vs treatment research studies, is limited. Elucidation of these factors could ease recruitment and retention resource burdens of researchers, safeguard the integrity of health-related intervention studies, facilitate participant inclination to access interventions, and ultimately enhance public health efforts [25, 26].

Despite the critical importance of successful recruitment and retention to study integrity as well as the resource-intensive nature of these activities [27, 28], studies reporting recruitment and retention strategies, as well as factors associated with successful recruitment and retention of study participants in health-related interventions remain rare, particularly for health and obesity prevention programs [27, 29–35]. To address this gap in the literature, this article aims to retrospectively look at the recruitment and retention marketing plan used in the HomeStyles RCT and discuss factors associated with outcomes of each component of the intervention.

## Methods

### Recruitment and retention marketing plan

**Fig. 1:**
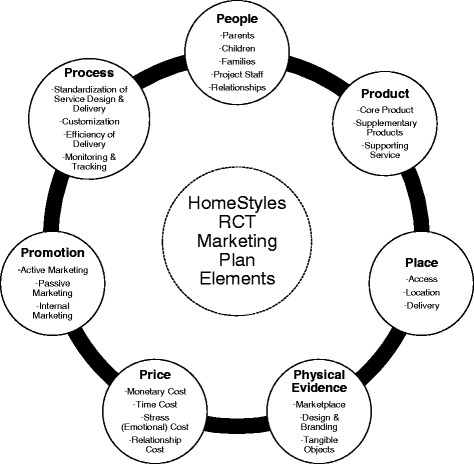
HomeStyles randomized controlled trial (RCT) marketing plan elements [37]

To meet recruitment and retention challenges, the HomeStyles project staff created a comprehensive, systematic plan using varied and multiple strategies [22, 36] based on the seven Ps of services marketing: people, place, product, physical evidence, price, promotion, and process (Fig. 1) [37]. The plan was frequently reviewed and adjusted to be responsive to new opportunities and technologies. The marketing plan began with an in-depth review of the literature to catalog effective marketing practices for health behavior interventions (e.g., Treweek et al. [35], Nicholson et al. [38], Page and Persch [24], Schoeppe et al. [23], and others [29, 31, 39, 40]) and input from the HomeStyles expert advisory board, community partners, and the target audience.

### People

The first P, people, had been clearly determined prior to the start of the study—parents who were the primary household food gatekeepers, were 20–45 years old, lived in the catchment area (Arizona or New Jersey), and had at least one child aged 2 to < 6 years, at least basic English or Spanish reading skills, and regular Internet access. Parents were the primary target, but their preschool children and family unit also were considered “customers.” Thus, it was critical to be certain all of them gained satisfaction from the “product” (a healthier, happier family delivered via the HomeStyles program).

Project staff also were part of the people mix because they were responsible for establishing and maintaining relationships with parents, either remotely through the program website and printed materials or in actuality via phone, SMS (texting), and email. To support recruitment and retention [41–44], bilingual, culturally sensitive project staff (HomeStyles specialists) were trained in customer service. The specialists were trained to rapidly respond to participant queries coming in by email or the dedicated toll-free line and use a positive, nonjudgmental, courteous, “can do” tone. Scripted responses were created to ensure accuracy and consistency of information proffered and equal handling across treatment groups.

### Product

Product, the second P, was a major component of this overall project, taking over 2 years to create the core products (i.e., instructional materials). The development of HomeStyles is described in detail elsewhere [2–6]; a brief summary follows to provide context for this paper. HomeStyles is an online program designed to enable and motivate parents of preschool children to make quick, easy, no-cost childhood obesity-preventive changes to their home environments and family lifestyles. This program has a social ecological framework [45], is grounded in Social Cognitive Theory [46, 47] and Adult Learning Theory [48–53], and uses motivational interviewing principles [54–57]. Content was developed to be responsive to the latest research findings and childhood obesity prevention recommendations [1, 58–61]. All instructional and data collection materials were thoroughly pretested with members of the target audience to ensure a high degree of comprehension, relevance, usability, interest, preference, attractiveness, and satisfaction [2–5, 62–64].

HomeStyles materials and study procedures were methodically and carefully developed with input and buy-in from parents at every step because considerable evidence suggests participant perceptions of program characteristics (e.g., content, packaging, promotion, time commitments) affect whether individuals choose to enroll and continue in a study [65]. A common reason families do not participate in studies is that they dislike the idea of being the subject of research [65]. Accordingly, care was taken to avoid using terms such as “subject” (“parents,” “families,” or “participants” were substituted) and “research” (“study,” “program,” or “project” were used instead) as well as terms implying “schoolwork” (e.g., instead of “work on a lesson,” participants “get to review a guide,” or instead of “take the posttest,” parents “get to go to the survey café”). Treatment groups were not referred to as “control” or “experimental” (rather, “safe HomeStyles” and “healthy HomeStyles”), because it was important to keep participants blind to their treatment group, as well as to avoid research connotations, disappointment that may occur with assignment to the control group, efforts by the control group to seek alternative access to experimental group materials, and/or differential dropout rates [66–79]. This method of referring to treatment groups also served as a constant reminder to staff that this RCT had two analogous treatments differing only in content.

Parents who completed an online screener and met eligibility criteria were immediately invited to participate and complete the informed consent. To facilitate recruitment, the informed consent was visually appealing, clearly written using common language, and easy to complete and submit [23, 80–82]. Participants who consented and finished the baseline survey were systematically assigned by computer to a treatment group. Participants could immediately access instructional materials after group assignment with the aim of limiting attrition [83]. Experimental group parents received web-delivered instructional materials focused on weight management-related topics (i.e., diet, physical activity, sleep) and the attention control group received a bona fide intervention with materials covering home safety topics. Both treatment groups had access to a series of 12 instructional guides in a 4-page minimagazine format. All parents received the same first guide (specific to their treatment group), which provided an overview of the intervention and tips for deciding which subsequent guides would be the best match for their families’ goals. With the exception of the first guide, parents could select any guide in any sequence. Progress through the RCT was as follows:

- Level 1: Baseline survey
- Level 2: Receive overview guide and then every ~ 16–30 days, choose a different guide for a total of four guides, then complete the midpoint survey
- Level 3: Every ~ 16–30 days, choose a different guide for a total of four guides, then complete the post survey
- Level 4: Choose a different guide or revisit a previously chosen guide, and ~ 30–60 days later, complete the follow-up survey
- Level 5: Choose a different guide or revisit a previously chosen guide, and ~ 30–60 days later, complete the long-term follow-up survey

Completion of the entire RCT was estimated to take 12–18 months [6].

Considerable effort also was spent on creating study surveys that were psychometrically sound as well as accurately understood, enjoyable, and easy to complete by the target population [6, 62–64, 84–86]. Research shows that participant enjoyment of data collection procedures, especially the first round of data collection, contributes favorably to participating in future data collection [41, 85, 87]. Thus, all HomeStyles RCT data collection was conducted online at a time of the participant’s choosing. Survey design strategies to help reduce participant burden included arranging items with the same answer choices (e.g., strongly agree to strongly disagree) in a matrix with alternating shading to increase reading ease as well as speed and accuracy of completion; using font treatments (e.g., bold, color, italics) for emphasis to promote rapid comprehension; including brief, clear instructions for completion; varying clip art page headers and question types to promote interest; limiting the number of items appearing at one time to minimize the need to scroll the page; using primarily radio buttons and drop-down boxes with few items that required free text answers; using adequate white space to decrease visual burden and give a feeling of rapid progress; and giving mental breaks to prevent fatigue (e.g., periodic placement of a photo evoking relaxation and calmness, such as a beach, with a caption encouraging the participant to take a deep breath and think about the fresh scent of the ocean). In addition, the online surveys were thoroughly pretested and refined, then pilot-tested and refined to ensure there were no technical issues that would frustrate participants. Pilot-test participants (*n* = 550) rated overall satisfaction with the survey as 4.5 out of 5 points.

Supplementary products were developed to enhance the value of the core products. These included tracking forms coordinated with instructional guides that promoted goal setting and monitoring of progress toward goals, mailed enhancements that facilitated application of the guide content (e.g., measuring cups to support portion control, cutting board to encourage fruit/vegetable intake), and extra guide-specific information and resources on the website (e.g., tips, goal ideas, links to helpful external videos and websites).

Supporting services offered were the friendly HomeStyles specialists who were readily available by toll-free phone or email. The services offered included technical problem solving (e.g., website login), providing additional facilitative information, and encouragement.

### Place

The third P, place, was decided a priori. HomeStyles was delivered online because nearly all U.S. families have Internet access [88] and use it often to gather health-related information [89–91]. The online delivery also permitted parents to access the product at times and locations convenient to them, thereby affording flexibility and giving parents control over participation, which is associated with increased retention [92]. An advantage of online over in-person interventions is that it overcomes transportation and child care issues frequently cited as barriers to participant retention [92]. Additionally, online access is cost-effective, and it offers an excellent probability of enhancing the ability to economically sustain the availability of project materials after grant funding ends. To ensure participants had quality experiences with the project website, it was professionally designed and developed, evaluated by the target audience, and intensively tested to confirm it functioned as intended and was bug-free.

### Physical evidence

The fourth P, physical evidence, includes the marketplace itself, design of materials, and tangible objects. The “marketplace” for HomeStyles was the website where the service was delivered as well as email, text, and phone where staff and participants interacted. It also included the recruitment materials that led potential participants to the project website. A project identity and design program was carefully planned in collaboration with a professional graphic designer and iteratively tested with parents of preschool children and then refined to confirm that parents found it eye-catching, family-friendly, positive, appealing, and suitable. All project materials (e.g., guides, trackers, enhancements, website, envelopes) were branded with the project logo to create ready recognition of project-related communications and a visible reminder of HomeStyles to participating parents [41]. Project logos also confer a sense of study legitimacy and visibility that are helpful for both recruitment and retention [93]. In addition, all printed materials (e.g., recruitment cards, copies of guides mailed to participants with enhancements) were printed professionally on glossy, heavy paper stock to evoke a feeling of quality and reduce chances of materials being thrown away [94].

### Price

Price, the fifth P, centers on “costs” to the participants. Although there was no dollar cost for parents associated with participation, there was a time cost as well as the potential cost of stress on family relationships associated with participation (e.g., stress associated with devoting time to the intervention that would otherwise be used differently, setting and working toward goals, and coping with possible family resistance to change). Time commitments are an important determinant of participation [65, 95]. To address time costs, recruitment materials stated there were 12 guides that each took about 15 minutes to review, that families would spend a few minutes daily making simple changes to help their families, and that total length of time for the intervention was about 12 months. Materials also indicated the changes were easy to make and could fit into busy schedules and tight budgets. Additionally, recruitment materials and reminders to participants stated that parents would receive stipends for completion of study surveys as well as gifts to help them implement simple changes.

Stipends were offered for two reasons: to compensate participants for the time spent completing surveys (i.e., working for the researchers) and because stipends are associated with improved recruitment and retention success [36, 87, 92, 96–100]. Cash stipends were paid after completion of each survey, and as commonly done in other studies, amounts increased modestly with each subsequent survey [41]. “Gifts” took the form of guide enhancements (e.g., measuring cups) that not only served to facilitate application of guide concepts but also helped to forge and renew relationships between participants and researchers, gently remind parents about participation in HomeStyles, and build goodwill. Other tokens of appreciation and reminders to participate included holiday cards, refrigerator magnets, and key chains, all displaying the HomeStyles logo. Anecdotal evidence of participant appreciation of stipends and gifts included unsolicited emails from parents that told project staff how they planned to use stipends to benefit their families and about the excitement of their children when packages with the project logo arrived in the mail.

To promote frequent visits to the website and introduce an element of fun and, thus, promote retention [41], parents had the opportunity approximately every 10 days to earn a “bonus buck” ($1 US) that would be added to their next stipend. The bonus bucks asked parents to answer an interesting or fun question (“If you wrote a song about HomeStyles, what would you call it? Who would you get to sing it?”; “CNN is on the phone—what would you tell them about how HomeStyles helped your kids?”)

To lower stress costs, recruitment announcements specified that a friendly HomeStyles specialist was available by phone or email. There was a “stress busters” and “confidence builders” section placed outside the secure login area of the project website so that potential participants could review it while deciding whether to participate. Similarly, to minimize possible relationship costs parents might encounter from potentially resistant family members, a “get more” section of the project website outside the login area encouraged parents to make this a family project and get everyone involved.

To promote retention [42, 92, 101], throughout the intervention parents received periodic reminders about the benefits and return on their time investment (e.g., happier, healthier families); stress reduction suggestions (e.g., “make it a family agreement, not a family argument,” “if you get off track with your goals, just start over,” “choose another goal that may be easier”); encouragement to persevere (“keep yourself moving by thinking about how much you love your family and the steps you can take to keep them healthy”); and friendly, encouraging nudges delivered by email, voice mail, and/or text (per parent preference). When parents were eligible to complete a survey, friendly announcements reminded them of the opportunity to earn the associated cash stipend.

Another aspect of price for RCTs are expenses associated with recruitment and retention of participants. These included monetary costs (e.g., printing and distribution of recruitment and other study materials), staff costs (e.g., energy and time allocated to training and recruitment), and relationship costs (e.g., effort to maintain relationships with existing community partners and attract new ones). An additional cost was the stress costs associated with the intensity of recruitment and retention activities, slow accrual of enrolled participants, and loss to follow-up despite intensive staff efforts. To keep these costs in check, the research team carefully planned recruitment and retention activities and identified an array of methods to keep monetary costs under control. For example, professionally designed recruitment materials were distributed in print form only in high traffic areas likely to reach the target audience (e.g., pediatrician’s offices; child care centers; Women, Infants, and Children program [WIC] offices), paid advertising was kept to a minimum, in-kind marketing opportunities were sought (e.g., radio interviews, links from other websites), and the bulk of recruitment occurred electronically.

Project staff stress was managed by actively involving staff in recruitment and retention decisions; keeping communication open and positive; clearly communicating procedures and adjusting them as needed to be responsive to pertinent events, observations, and opportunities; rotating duties; keeping everyone up-to-date on progress and complimenting efforts; holding refresher trainings; giving staff feedback and recognition; holding occasional staff appreciation events; and maintaining a high level of staff enthusiasm for the project. These efforts fostered cohesion and a strong team culture, which paid off, as evidenced by a continual positive workplace atmosphere and low staff turnover (primarily students who graduated).

### Promotion

The sixth P, promotion, addressed all forms of marketing HomeStyles to prospective participants. The content of the recruitment marketing materials was informed by qualitative data collection activities (i.e., focus groups [*n* = 139] and cognitive testing of intervention materials [*n* = 512]) conducted as part of the overall project formative research and supplemented with quantitative preference surveys with English- and Spanish-speaking parents of preschool children residing in New Jersey and Arizona [2–5]. Salient findings from the formative research relevant to recruitment materials included parents’ strong dislike of the terms “obesity” or “overweight” and messaging that implied a need to organize or “get things under control” [3, 4]. Similarly, others have reported that parents tend to have little concern or interest related to obesity [23, 33].

Parents preferred messages that projected happiness, fun, and quick and unique solutions to everyday challenges. They liked attention-catching colors, appreciated photos of families of varied races/ethnicities, were curious about other families’ behaviors and opinions for self-comparison purposes, valued other parents’ endorsement of HomeStyles, and had a robust desire to build stronger bonds with their children. In fact, their desire for happier, closer family relationships ranked higher in importance than improving the health of their children [5]. Thus, the content and design of all HomeStyles materials, including recruitment materials, aimed to appeal to parent preferences. Recruitment materials were cognitively tested with both English- and Spanish-speaking parents and parent educators to confirm the clarity of the content, the appeal of the content and design, and the likelihood that parents would respond by visiting the website to sign up for or learn more about the study [2].

Recruitment materials took a wide array of forms and were simultaneously disseminated in a variety of ways to extend the reach and speed the accrual of participants [22, 36, 83, 102]. Nearly all recruitment was done in a passive form (e.g., posters, flyers) to contain costs and because this method was well suited to an online study. Additionally, the limited research comparing active with passive recruitment indicates that either form can work, and participants recruited by these different methods usually do not differ by baseline demographics, psychosocial variables, or attrition [103].

Passive marketing efforts included distribution or display of printed materials (e.g., flyers, bookmarks, posters, magnets, key chains) by community partners (e.g., pediatricians’ offices, fitness centers, schools, preschools/day care centers, workplaces, community centers, health fairs, and farmers markets). To help existing community partners learn more about HomeStyles and to build new partnerships, project staff directly marketed HomeStyles to them via webinars held at convenient times, in-person visits to partners’ offices, brief talks at professional meetings, and short informational YouTube videos specifically tailored to key recruitment partners (i.e., registered dietitians, pediatricians, and early childhood educators). To ensure that community partners remembered HomeStyles, researchers made at least two personal follow-up calls or contacts to encourage them to promote HomeStyles to their clients, confirm receipt of the recruitment materials sent, and answer questions.

For electronic recruitment announcement distribution, an inventory was created and regularly updated that included addresses of listservs that could reach targeted participants directly or through trusted sources such as workplaces, religious groups, philanthropic or community organizations, preschools/day care centers, professional associations, and extracurricular activity groups. Listserv administrators were contacted by email and/or phone to encourage them to forward the electronic recruitment announcement to listserv recipients. Administrators had the opportunity to contact researchers via email or phone to learn more about the project. Whenever possible, supervisors of the listserv administrators were contacted to gain their endorsement for forwarding the emails. Listserv administrators were contacted three or more times over the recruitment period. Appeals to listserv administrators were sent from an official university email address and included university logos to establish credibility. A professional study recruitment agency also was employed to distribute electronic announcements about HomeStyles to the members of their research panel.

Other electronic recruitment venues included notices posted to websites that target parents, online local newspapers, local businesses, and parenting-related blogs. Social media also was used for recruitment, as recommended by others [104], although it was not possible to create online communities for parents to interact with each other, owing to the need to maintain participant blinding to treatment group, prevent contamination (i.e., sharing of knowledge with parents in the other treatment group), and protect participant privacy. By adjusting security and privacy settings, it was possible to create Facebook® (Menlo Park, CA, USA) and Pinterest® (Cold Brew Labs, San Francisco, CA, USA) pages for parents to learn about the study and link these pages to the study website to facilitate enrollment.

Print media provided gratis was used as a recruitment tool, though in a limited way. A university-based collaboration with a children’s cooking magazine afforded an opportunity to include an announcement about HomeStyles in copies distributed in New Jersey. There was some success with product placements in printed newspapers, which involved mostly interviews that led to brief articles about the project with information on how to sign up. Owing to lack of access and budgetary limitations, recruitment efforts using radio and television were limited to brief interviews.

Paid advertising was purchased through Facebook® because of its widespread use and potential to reach parents. A series of 64 ads were created, all of which included a photo of a preschool child and text promoting HomeStyles. The ads systematically varied the photo (four races/ethnicities, two sexes), salutation (Hey Dads!, Hey Moms!, Hey Moms & Dads!, and Hey Parents!), and closing statement (Click here! and Find out how!). Four evaluators unanimously agreed that all eight photos had similar lighting and clarity and depicted children of a specific race/ethnicity, sex, and age who directly faced the camera and had a “happy” expression. Facebook® criteria were set to target ads to parents aged 20–45 years with children aged 0–12 years who were interested in happy kids or fitness and wellness. These ads ran for 250 days [105].

Some direct, active recruitment marketing was conducted at community events, parent resource centers, WIC offices, and farmers markets. Internal marketing efforts overlapped with project staff stress management procedures previously described. In addition, another important component of the recruitment campaign was to exhaustively rally project staff’s families, colleagues, friends, and neighbors to distribute recruitment materials and share the word about HomeStyles. In addition, these individuals contributed ideas for new recruitment methods and opportunities.

To keep participants blind to their treatment group assignment, it was critical for all recruitment materials to be applicable to both groups. To achieve this, the study expectations for the experimental and action control group were held constant. In addition, the program was described as one that would help kids be happier, healthier, and safer—terms that were applicable and true for both treatment groups.

### Process

The final marketing P, process, is focused on ensuring standardized, customized, and efficient service delivery. The website helped to ensure that service delivery would be standard across participants. Because not all transactions were web-based, standard operating procedures (SOP) and manuals were created and implemented to promote quality control and ensure all staff performed them uniformly. These SOPs included templates (e.g., scripts, email text) for responding to commonly asked participant questions, procedures for handling technical problems, and complete and illustrated instructions for preparing all participant mailings (e.g., enhancements, holiday cards). In addition, staff had a clear chain of command for guidance for handling problems that did not have an SOP or when they felt the typical SOP needed to be customized to better meet a participant’s needs.

Timely product delivery and responses to parent queries were top priorities. To address this and improve retention [38], the project had a dedicated email account for participants and a toll-free phone line that was staffed consistently and from which calls were responded to quickly. Given that providing reinforcement soon after completing RCT activities helps promote retention [41], parents were sent their stipends electronically in the form of a gift card for a store of their choosing within 2–3 days of completing a survey. Likewise, soon after selecting a new guide, staff mailed a printed copy of the guide and, for at least every other guide mailing, included a supporting enhancement.

Another component of process is to monitor marketing activities and track progress toward enrollment and retention goals. The research team used Excel software (Microsoft, Redmond, WA, USA) to create recruitment-tracking spreadsheets that organized participants by group (experimental, control), geographic location (New Jersey, Arizona), and language (English, Spanish). Plotting spreadsheet data by group × location × language enabled researchers to assess progress, adjust allocation of recruitment resources (e.g., staff time, recruitment flyer printing and distribution, recruitment activities), and forecast when recruitment goals would be reached. Recruitment reports were generated and reviewed twice weekly to facilitate timely, informed research management decisions [22]. In addition, these reports were scrutinized to cross-check data to detect and eliminate duplicate completions of the baseline survey from recruitment counts, a problem noted by others [86, 106].

Creation of similar spreadsheets supported participant retention efforts. The website tracked participant use of the website, enabling researchers to download reports of activities that were organized in spreadsheets. Retention spreadsheets tracked each participant’s progress through the RCT and days elapsed since his or her last communication with project staff or website. Charting retention data permitted researchers to visualize participant progress throughout the time course of the RCT, monitor the rate at which participants completed each aspect of the RCT, and rapidly intervene when participants appeared to be experiencing barriers that may cause them to become inactive and drop out. The retention-tracking spreadsheet was annotated to indicate when and how inactive participants were contacted. For instance, when retention reports indicated a participant had not been to the study website for 30 days, staff contacted the participant via phone and email about every 10 days to encourage them to return to the website and continue participation. After 60 days of inactivity, staff reviewed the participant’s previous guide choices, mailed a new guide, and set the website to send the guide-specific nudges. This procedure was repeated until parents had completed all guides associated with a level, at which time staff contacted inactive parents by phone, email, and mail (using branded, bright blue, shiny Mylar envelopes [DuPont Teijin Films, Chester, VA, USA]) to encourage them to complete the next survey. As noted by others [35], staff anecdotally observed that phone calls tended to result in participant action more so than emails. Staff were patient and persistent in their intensive efforts to retain participants, calling, emailing, texting, sending messages via Facebook® as needed to reach them and encourage continued participation [23, 92, 107]. Retention reports were generated and reviewed twice weekly to facilitate timely contacts with participants as well as retention [92] and reassessment of recruitment goals.

## Results and discussion

### Recruitment and retention marketing outcomes

Parents signed up to participate in the RCT on a rolling basis, with the recruitment time line responsive to enrollment, retention, and study time line. Originally, a 24-month recruitment period was planned to recruit ~ 300 English-speaking and ~ 300 Spanish-speaking families, with the goal of retaining ~ 60% (i.e., 210 per language) of them for the duration of the study. Owing to a series of natural disasters that delayed project development and therefore set back the start of recruitment efforts, coupled with the inability to extend the grant period more than 1 year, the recruitment period had to be shortened to 15 months. Thus, recruitment activities commenced intensively, which involved concurrently distributing recruitment materials via numerous channels.

Throughout the recruitment period and for 12 months after recruitment, a study goal was to retain participants. Retention efforts are critically important to long-term studies such as HomeStyles. Premature loss of participants can diminish internal and external validity and prolong trials if additional participants need to be recruited [24, 93]. Additionally, differential attrition may bias study outcomes [24]. Like recruitment, published literature on retention of intervention participants remains scant. However, unlike the lack of an a priori mechanism for assessing HomeStyles recruitment efforts, strategies for assessing retention efforts were planned. The results of recruitment and retention efforts are presented using marketing outcome indicators of profitability and progress.

### Profitability

Profitability is the goal of marketing efforts. That is, what is the payoff that results from all the marketing efforts? HomeStyles was designed as an intervention study rather than solely testing the effectiveness of recruitment and retention strategies. Nonetheless, it is possible to explore several aspects of recruitment and retention outcomes retrospectively, as well as prospectively with data derived from paid Facebook® advertising.

**Fig. 2:**
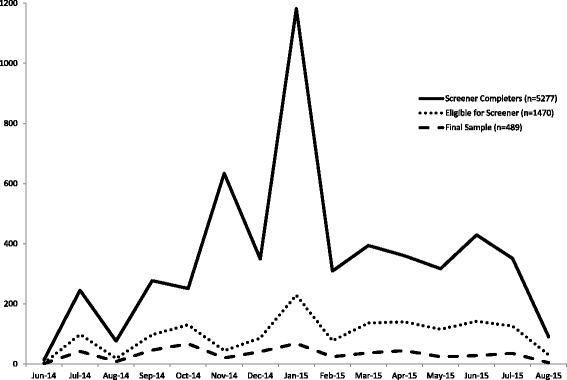
Randomized controlled trial recruitment: individuals completing study eligibility screener, eligible participants, and final sample

Figure 2 shows the rate at which participants responded to recruitment materials and completed the online screener. Overall, 5494 individuals visited the screener website and 5277 completed it, indicating that the marketing plan was effective at generating sufficient interest to draw potential participants to the screener. With the exception of a peak in December 2014/January 2015, when the professional study recruitment agency began distributing electronic announcements about HomeStyles to the members of their research panel, it is not possible to discern the contribution of individual recruitment methods. However, to gain some insight, a minisurvey was sent to those completing the long-term follow-up survey to retrospectively gather this data. As shown in Table 1, many recruitment sources were identified with day care providers/preschools, website postings, emails, and friends being the most commonly named sources. Most (81%) participants found out about HomeStyles from a single source.

**Table 1:** HomeStyles recruitment (*n* = 112)

	All participants (*n* = 112)	
How did you hear about HomeStyles?	No. of participants	Percent
Friend	18	16.1
Family	4	3.6
Coworker	9	8.0
Home visitor	6	5.3
WIC office	4	3.6
Daycare provider/preschool	21	18.8
Rutgers researchers	1	0.9
Email notice	19	17.0
Website posting	18	16.1
Paper flyer poster	5	4.5
Social media (e.g., Facebook®, Twitter)	8	7.1
Not sure, do not remember	16	14.3

Facebook® ads ran between June 2014 and February 2015. The 3,800,985 impressions and 100,603 potential participants reached generated 2639 visits to the HomeStyles screener website (48% of all visits) and 6 parent enrollments in the study. Fisher’s exact test revealed no difference in effectiveness of the ads by composition of their components (i.e., child race/ethnicity, child sex, salutation, or closing). A systematic review of health research using Facebook® ads to recruit adolescents also indicated varying success—a comparison of recruited adolescents to clicks on paid advertisements was 2–3% for four of the five studies reviewed, with researchers in a single study reporting a 72% recruitment rate [30]. Similarly, Facebook® was found to be an ineffective and costly recruitment method for young adults and smokers, despite its wide reach [27, 108].

Only 39% (*n* = 1470) of the screener completers met RCT eligibility criteria, suggesting that recruitment materials were not sufficiently specific to the target audience. The most common reasons for ineligibility were parent and child ages not within the study age range: 15% of parents were not aged 20–45 years, and 57% of respondents did not have at least one child aged 2 to < 6 years. Other reasons were residence outside the catchment area (49%), parent not being the family food gatekeeper (17%), and parent not having regular Internet access (5%). A majority of those ineligible did not meet one (65%) of the eligibility criteria; the remainder did not meet two or more criteria. Although recruitment was actively conducted only in the catchment states, the widespread use of the Internet to recruit likely contributed to the high rate of ineligibility due to parents’ residences being in other states. The high percentage of children outside the study age range may be reflective of language used in the recruitment materials (i.e., “young children”). With regard to ineligibility due to child age, 22%, 14%, 35%, and 10% had children younger (<2 years), somewhat older than the study age range (6 to < 9 years), much older (> 9 years), or a combination of children younger and older than the study age range. Interestingly, 19% had no children living in the household. A more explicit statement of children’s ages may have reduced unnecessary traffic to the screener; however, the decision to keep the description somewhat vague was made a priori to limit participation to only parents explicitly indicating that their children were in the targeted age range.

Of those eligible to participate, 34% (*n* = 507) gave informed consent and completed the baseline survey. After elimination of participants with unconfirmed addresses and implausible answers, the final sample equaled 489, a final capture rate of 33% of those eligible to participate. HomeStyles recruitment outcomes are comparable to those of other studies. For instance, a recent review reported that the typical capture rate for obesity prevention and treatment trials targeting minority or low-income children ranged from 10% to 90% [33]. A capture rate toward the lower end of the typical range may be due to the length of the HomeStyles RCT.

Had it been possible to extend recruitment for HomeStyles to the full 24-month period and maintain the average monthly accrual rate of ~ 33 eligible, consenting, participating parents (489 parents/15 months), enrollment would have exceeded the original goal (33 parents/month × 24 months). This outcome would place this RCT in the minority of studies reporting on time recruiting success—authors of a review of 114 multicenter trials reported that less than one in three achieved their recruitment target within the originally planned time frame, and another one-third extended recruitment to meet goals [29].

Recruiting and retaining minorities has been a long-standing challenge for researchers, for many reasons [43]. The racial/ethnic distribution of recruited participants was diverse, with 58%, 23%, 8%, 4%, 2%, and 4% being white, Hispanic, black, Asian Indian, Asian, and other (e.g., Pacific Islander, American Indian, Alaskan Native, or mixed race), respectively. This may be reflective of the care taken during the development of intervention materials to choose photos clearly depicting children and families from all racial/ethnic backgrounds and to use culturally sensitive language. The high rate of recruitment of Hispanic participants is notable because of the many barriers typically cited that prevent their participation in research studies [43]. One commonly named barrier is lack of culturally competent research. To overcome this, the Spanish-language versions of the HomeStyles materials were developed using in-culture translations by a professional translation team that also had expertise in health and nutrition. Additionally, the translated HomeStyles materials underwent cognitive testing with Spanish-speaking parents who originated from an array of Spanish-speaking countries and then were refined to improve clarity and understanding across regional variations of the Spanish language. The project staff also included many bilingual, bicultural individuals from many different Spanish-speaking countries located in Central and South America.

About one-fourth (23%) of Hispanic participants chose to use the Spanish-language version of the HomeStyles materials, well below the demand anticipated. Although Spanish is the most-spoken language after English in the United States [109], the National Survey of Latinos indicates that 5% of U.S.-born Latinos speak only Spanish, whereas 60% of foreign-born Hispanic adults in the United States speak only Spanish [110]. Speaking a language does not necessarily mean a person can read or write it. Among bilingual speaking Hispanic adults in the United States, about one-fifth prefer reading instruction manuals and newspapers in Spanish [111, 112]. The ratio of Hispanics in the RCT choosing to use Spanish vs English materials was comparable to those preferring to read instructions and newspapers in Spanish. Of those who chose Spanish HomeStyles, 71% were foreign-born.

Use of the Spanish-language version of the HomeStyles materials was low, but the Hispanic enrollment overall was substantially higher than the proportion (i.e., 17%) comprising the U.S. population [113]. Factors contributing to the success in recruiting Hispanic participants may be due to the culturally sensitive Spanish recruitment materials, the availability of Spanish HomeStyles guides, and the inclusion of pictures of Spanish children and families in project materials conveying a feeling of inclusiveness and personalization. Another contributing factor may be recruitment efforts in areas densely populated with Spanish families and the fact that each of New Jersey and Arizona has approximately 2 million Hispanic residents, among the highest in the United States [114]. Future researchers should investigate how these and other factors influence recruitment of Hispanic audiences [43].

### Progress

After recruiting and enrolling participants, the goal is to facilitate and support their progress through the RCT (i.e., encourage them to be “repeat customers”). There were two strategies for evaluating retention efforts: tracking progress and retention efforts and identifying factors associated with study completers vs noncompleters.

#### Tracking progress

The first strategy was to track progress and, as described previously, quickly intervene with friendly reminders from project staff via phone, email, and texts. Additionally, these data were used to identify key points at which participants left the RCT. Table 2 shows the RCT activities and number of participants remaining at each point. The attrition rate at the midpoint survey compared with the baseline survey was 46%. Similar comparisons of each subsequent survey administration with the immediately preceding survey indicated that attrition rates steadily declined to 35% (midpoint vs postsurvey), 18% (postsurvey vs follow-up), and 12% (follow-up vs long-term follow-up).

**Table 2:** Retention at each time point in the randomized controlled trial and days to completion

Time point	All participants	Experimental group					Control group				
	n (%)	Total experimental n (%)	Active partici-pants^a^ n (%)	Days to next time point Mean ± SD	Passive partici-pants n (%)	Days to next time point Mean ± SD	Total control n (%)	Active partici-pants^a^ n (%)	Days to next time point Mean ± SD	Passive partici-pants n (%)	Days to next time point Mean ± SD
Baseline survey	489 (100)	252 (100)	140 (55.56)	189.14 ± 138.72	112 (44.44)	402.76 ± 130.9^b^	237 (100)	124 (52.32)	169.15 ± 123.6	113 (47.68)	380.42 ± 113.75^c^
Mid-point survey	264 (53.99)	140 (55.56)	126 (90)	197.36 ± 108.93	14 (10)	336.86 ± 95.66^b^	124 (52.32)	102 (82.26)	163.64 ± 94.91	22 (17.74)	309.89 ± 19.99^c^
Post survey	172 (35.17)	89 (35.32)	86 (96.63)	43.46 ± 33.36	3 (3.34)	140.33 ± 43.16^b^	83 (35.02)	80 (96.39)	45.53 ± 36.95	3 (3.61)	95.67 ± 25.11^c^
Follow-Up survey	141 (28.83)	70 (27.78)	70 (100)	55.43 ± 63.10	0 (0)	0 (0)	71 (29.96)	71 (100)	51.16 ± 40.44	0 (0)	
Long-term follow-up survey	124 (25.36)	61 (24.21)	--	--	--	--	63 (26.58)	--	--	--	--

^a^Active participants were defined as retrieving 3 of 4 possible guides on their own from the website whereas Passive participants were defined as retrieving less than 3 of 4 possible guides on their own from the website between the following time points: baseline and mid-point survey; mid-point and post survey. At all other time points, participants were defined as Active if they retrieved 1 of 1 possible guide on their own and Passive if they did not retrieve a guide on their own from the website

^b^Independent *t*-tests and Mann Whitney U tests indicate significant (p ≤ 0.05) differences of active and passive participant days to next survey point in the experimental group

^c^Independent *t*-tests and Mann Whitney U tests indicate significant (p ≤ 0.05) differences of active and passive participant days to next survey point in the control group

The number of individuals prematurely leaving the RCT was similar in both treatment groups, indicating that attrition was not differential. This also suggests similar satisfaction with the intervention materials and overall participation for both groups [44]. In fact, there were few satisfaction differences in parents in the experimental group (*n* = 59) vs the control group (*n* = 53) who completed the satisfaction survey (*n* = 112) administered after the long-term follow-up survey. All reported high satisfaction using a 1–5 scale (strongly disagree to strongly agree) on guide attractiveness (4.32 ± 0.87 SD), interestingness (4.40 ± 0.65), usefulness (4.51 ± 0.60 SD), ease of use (4.57 ± 0.50 SD), and helpfulness to family (4.41 ± 0.67 SD), indicating the program itself supported their completion of the program. The only between-group difference in satisfaction was that the experimental group rated the attractiveness of the guides higher than the control group did (4.53 ± 0.50 SD vs 4.09 ± 1.11 SD, *df* = 110, *p* = 0.012).

Table 2 also compares “active” participants—that is, those who went to the project website on their own to retrieve most of the study guides with those who were “passive”—those for whom the project staff had to choose most new guides for them. A comparison of within-group differences using independent *t* tests and Mann-Whitney *U* tests for both experimental and control groups revealed that active participants progressed through the study time line significantly (*p* < 0.05) faster than passive participants. Days to the next time point for survey completion did not differ between groups for active or passive participants for any survey time point, except at the midpoint survey, when active control participants spent significantly (*p* = 0.043) fewer days getting to the next time point than active experimental participants (163 vs 197 days). Additionally, chi-square tests revealed no significant differences in the proportion of active and passive participants at any time point between treatment groups. Not surprisingly, the passive participants rapidly declined in number after the midpoint survey because many left the study before this data collection point. Their passive behaviors suggest lack of engagement with the RCT early in the time line.

The high rate of attrition at the second data collection point followed by slower attrition has been reported by others [41, 86, 93, 115]. Furthermore, the 25% overall retention rate at the long-term follow-up was comparable to or greater than the rates reported by many others for online interventions. For instance, an online lifestyle behavioral intervention retained 22% of the original sample at the 24-month follow-up [116], and an online depression program trial retained 23% of the original baseline sample. Researchers using other Internet interventions have reported lower retention rates, such as 1% in a 12-week panic disorder self-help web program [117], 13% in a healthy lifestyle program [118], 15% in an online weight management intervention [119], and some higher (e.g., a nutrition education program for adults retained 48% of the sample at 4 months [120]).

Several researchers have suggested that the considerable rate of noncompleters of web-based health interventions may be due to the flexibility of these programs, anonymity and freedom of participants, ease of deciding to use the intervention “sparingly” or completely discontinue participation, lack of consequences for nonuse, and lack of immediate observable health benefits [32, 86, 121–123]. Although these reasons also can be applied to other types of nonmandatory, community-based interventions, determining how to increase participant commitment to completing online public health interventions remains important—especially with the increased use of the Internet as a source of health information and with the shrinking health education budgets that compel greater use of web-based interventions.

Table 2 also compares the retention rates of those who were actively participating (i.e., coming to the website on their own to choose their next guide) vs passive participants (i.e., staff selected and mailed a new guide to participants inactive ≥ 60 days). To promote retention, project staff demonstrated all the characteristics needed for successful follow-up: “persistence, ingenuity, creativity, and dedication” and a “high tolerance for frustration” [124]^, p. 403^ Despite all of the retention efforts, the average monthly recruitment accrual rate of ~ 33 eligible, enrolled participants at baseline declined to ~ 18, 11, 9, and 8 remaining recruited participants/month at midpoint, post-, follow-up, and long-term follow-up surveys, respectively. Or, stated on a daily basis, it took ~ 3.6 days to recruit one participant who successfully completed the entire study. Thus, at this rate of enrollment and retention, to have attained the overall goal of 420 participants at the last data collection point, recruitment efforts would have needed to last more than 48 months (4 years, at the monthly accrual rate of 33 participants) and enroll nearly 1700 participants at baseline (i.e., at the long-term follow-up retention rate of ~ 25%).

The recruitment index (days to recruit one participant who successfully completes the study) was 3.6 days in this study. Although a comparable recruitment index data for behavioral intervention trials such as HomeStyles could not be located, HomeStyles’s recruitment index is substantially lower than the rates reported by others. For instance, Blanton et al. needed 33.2 days to recruit one eligible participant who completed a poststroke physical rehabilitation clinical trial lasting 2 years [125]. Similarly, in functional dyspepsia treatment trials, researchers reported a recruitment index of 45.9–190.3 days to recruit successful completers with recruitment efforts occurring at more than 40 participating sites [126]. The recruitment index for the study reported here may be lower for several reasons: It was a prevention vs clinical trial, eligibility criteria opened participation to a wide array of participants, parents had full control over when and where they could complete intervention activities, and it may indicate recruitment materials were convincing and appealing. Nonetheless, the recruitment and retention activities required intensive resource allocation, and the post hoc length of time calculated on the basis of retention figures that would have been needed to meet a priori retention goals was twice the generous 24-month recruitment period originally planned. These findings underscore the importance of allocating adequate project resources to recruitment and retention activities as well as allowing sufficient time to complete initial recruitment [126].

#### Predicting completers and noncompleters

The second strategy for evaluating retention was to determine factors predictive of study completers with the goal of informing future research. Prior knowledge of characteristics that may increase attrition risk can help researchers target and tailor retention efforts. Predictors of attrition in RCTs remain understudied, and studies that do exist tend to be focused on sociodemographic characteristics (e.g., age, education level, income) with little attention to other factors that may affect whether individuals complete an intervention (e.g., health status, stress, family support). HomeStyles was predicated on the socioecological model because individual and family health are determined by multiple levels of influence, including intrapersonal, interpersonal, and environmental factors [127]. Thus, the relationships between retention and factors at each of these levels were examined using the RCT baseline data [6, 128] analyzed with SPSS version 24.0 software (IBM, Armonk, NY, USA).

Factor selection was guided by previous retention research findings [25, 26, 65, 129–139]. Intrapersonal factors included parent sex, race/ethnicity (white, Hispanic, black, or other), preferred language (English or Spanish), level of education, and paid employment outside the home. Other parent intrapersonal factors measured using valid, reliable scales, described in detail elsewhere [6], included their health status [140, 141], health-related quality of life [140, 141], depression severity [142], stress management self-efficacy [143], parenting skill self-efficacy, personal organization/consciousness [144], need for cognition (enjoyment of solving problems) [145, 146], healthy eating and physical activity outcome expectations, parent concerns about their child’s weight [147], and perceptions of parents’ own weights and their children’s weights [147]. Child intrapersonal factors included sex, health status [140, 141], health-related quality of life [140, 141], and problematic eating behaviors [148]. Interpersonal or family-level factors included number of children in the household, single- vs dual-parent household, family affluence level [149, 150], food security risk [151], family support for behaviors promoted in HomeStyles [152–154], negative child-feeding behaviors [155, 156], and family functioning [157–159]. Environmental factors included neighborhood safety [160], ease of accessing a large supermarket, and median family income derived from participant home address ZIP code [161–163]. A final factor included was whether the participant was recruited by the professional study recruitment agency’s panel.

Spearman rank-order correlation analyses confirmed that none of the factors were multicollinear (i.e., *r* < 0.50). Thus, all were entered into a forward conditional stepwise binary logistic regression analysis to identify factors significantly predictive of participant retention at each data collection point (midpoint, post-, follow-up, and long-term follow-up). As shown in Table 3, midpoint survey completers were 4.3 times more likely to be female and 1.6 times more likely to be white. Additionally, participants with more weight concerns for their child and less family conflict were significantly (*p* < 0.05) more likely to be midpoint survey completers. Postsurvey completers were 2.5 times more likely to be female and perceive their children’s health status to be better for each 1-unit increase on a 5-point response scale (very poor to very good). Follow-up survey completers were significantly more likely to perceive their children’s health status to be better and less likely to be restrictive of their children’s food intake for each 1-unit decrease on a 5-point response scale. Long-term follow-up survey completers were significantly more likely to be female and perceive their children’s health status to be better, and significantly less likely to be restrictive of their children’s food intake. These results remained the same even after including an interaction term between parent weight concerns for children and treatment group in the models. Predictors of retention did not differ between treatment groups either.

**Table 3:** Stepwise logistic regression analyses examining factors associated with survey completers (*n* = 489)

Overall models^a^	All participants				
	*B*	Wald *χ* ^2^	*p* Value	OR	95% CI
Model 1: midpoint survey completers^b^					
Race (white)	0.46	5.80	0.016	1.58	1.09 – 2.29
Parent sex (female)	1.46	11.89	0.001	4.31	1.88 – 9.89
Parent weight concerns for child	0.20	4.52	0.034	1.23	1.02 – 1.48
Family conflict	− 0.25	6.55	0.010	0.78	0.65 – 0.94
Model 2: postsurvey completers^c^					
Parent sex (female)	0.90	3.76	0.052	2.46	0.99 – 6.11
Perceived child health status	0.39	8.60	0.003	1.47	1.14 – 1.91
Model 3: follow-up survey completers^d^					
Perceived child health status	0.39	7.52	0.006	1.48	1.12 – 1.96
Child food restriction	− 0.23	4.05	0.044	0.80	0.64 – 0.99
Model 4: long-term follow-up survey completers^e^					
Parent sex (female)	0.47	4.21	0.040	1.60	1.02 – 2.50
Perceived child health status	0.47	8.75	0.003	1.60	1.17 – 2.19
Child food restriction	− 0.27	5.22	0.022	0.77	0.61 – 0.96

^a^Forward stepwise logistic regression analyses examining factors predictive of survey completion at each survey time point

^b^Completers (*n* = 264) vs noncompleters (*n* = 225)

^c^Completers (*n* = 172) vs noncompleters (*n* = 317)

^d^Completers (*n* = 141) vs noncompleters (*n* = 348)

^e^Completers (*n* = 124) vs noncompleters (*n* = 365)

Overall, those most likely to complete the HomeStyles RCT were females who perceived their children were healthier, were concerned about their children’s weight, and used positive child-feeding practices. These variables indicate intrapersonal and interpersonal level factors affected retention, but not environmental factors. Given the stresses associated with caring for less-healthy children and coping with household conflict, it is perhaps not surprising that parents with these characteristics were more likely to leave the RCT early. Self-selected participants, such as those in this study, tend to be drawn to studies that align with their interests—this may be the reason that those engaging in health practices promoted in this RCT (e.g., using positive child-feeding behaviors and concern for their child’s weight) tended to be retained. Female parents outnumbered male parents in this study by 14 to 1, which is the likely cause of the large CI shown in Table 3. Others have reported that some of these same factors were predictive of premature termination of participation in intervention studies, including illness [92, 164], family stress [165], and being male [120, 164], as well as many other factors that were not predictors in this study. For example, predictors of participant completers in other studies associated with behavioral intervention trials that were not predictors in this study include sociodemographic (e.g., household income, level of education, family size, race/ethnicity), parent intrapersonal (e.g., depression, management skills), child intrapersonal (e.g., perceived susceptibility of child to health risk), and family interpersonal (e.g., family support) characteristics, as well as neighborhood conditions [22, 24, 26, 33, 92, 129–134, 138, 164, 166, 167].

Researchers in numerous studies have cited barriers to continued participation associated with transportation, conflicts in scheduling study-related visits, inconvenient study site locations, and lack of child care [33, 92, 166]. However, owing to the online delivery of the HomeStyles intervention, these factors were irrelevant to study participant retention. Participant perceptions of the program (e.g., program content, packaging, time demands) also affect participation decisions [65]. As described previously, parents completing the satisfaction survey administered after the long-term follow-up survey (conclusion of level 5) reported high satisfaction with the program. Follow-up phone interviews with inactive HomeStyles participants (*n* = 48) revealed that participants enjoyed the materials, reminders, and program; however, key barriers contributing to their inactive status and attrition were time constraints, forgetfulness, and other personal reasons unrelated to the project that hindered them from staying active in the programs. Participants’ suggestions for keeping them active included more frequent reminders and more specific instructions on how to proceed with the next steps in the project.

## Conclusions

Our aim in this paper was to expand the scientific literature by providing a comprehensive review of the extensive and intensive recruitment and retention efforts employed in the HomeStyles RCT. Additionally, this is among the first papers to report recruitment and retention strategies for an online, community-based, parent-driven childhood obesity prevention intervention [116] and to report a comprehensive, systematic RCT recruitment and retention plan based on services marketing principles. Few studies have expanded the consideration of retention factors beyond sociodemographic characteristics and/or used theoretical underpinnings to guide the selection of retention factors when examining predictors of RCT completers [25, 26, 92, 101, 164].

Findings derived from the HomeStyles RCT highlight the need for far-reaching, concentrated, and varied recruitment strategies; sufficient time in the research plan for recruitment and retention activities; and creative, tireless, flexible, persistent project staff. Outcomes of recruitment and retention efforts also suggest shortcomings that, despite best efforts and use of the available research and advice on effective marketing practices for health behavior interventions [23, 24, 29, 35, 38–41, 168], still fell short of study goals. Clearly, prospective studies investigating recruitment and retention strategies that are firmly grounded in an a priori marketing plan, complete with budgetary allocations and time lines, are needed to advance the field of online health promotion program delivery and research [23, 33]. A continued lack of prospective as well as retrospective studies, such as the one reported here, will undermine the ability to fully establish the effectiveness of the ever-increasing availability of online self-help and prevention programs [33]. A deeper understanding of effective recruitment and retention strategies would permit wiser use of shrinking research resources while protecting the integrity of study execution and outcomes [22–24, 102].

## Abbreviations

RCT: Randomized controlled trial
SOP: Standard operating procedure
WIC: Women, Infants, and Children program
